# Ano-Vesicle Centre

**Published:** 1888-03

**Authors:** 


### ANO-VESICAL CENTRE.

A correspondent of the *Medical Press and Circular* writing from Vienna, says: At the last meeting of the Imperial and Royal Medical Society of Vienna Professor Rosenthal delivered an interesting lecture on the Ano-vesical Centre. It was known that the commencement of severe disease of the spinal cord was often preceded by paralysis of the sphincters of the bladder and the rectum. From this fact it was to be inferred with some probability that the centre of these organs was to be sought for in the spinal cord, though physiologists were not yet of the same opinion. Irritation of the spinal cord produced contractions in the bladder, and according to Budge the mechanism of the discharge from the bladder was a reflectoric one, and the course of the reflective act was to be explained in such a way that the sensible nerve fibres represented the centripetal parts, and the motory nerves the centrifugal parts of the reflective organ. This mechanism was furthermore dominated by nerve fibres from the brain to the spinal cord. Professor Rosenthal here referred to the contradictory statements of different authors as to the exact seat of the reflective centre in question. Some authors stated that the seat of this centre was below the lumbar plexus, others above it. This difference of opinions was to be explained by the fact that uncomplicated cases of this class were of a great rarity. Quincke, basing himself on the experiments which he had conducted together with Kirchhoff, and in which the spinal cord was compressed owing to fracture of the first lumbar vertebra, stated that the centre was to be sought for in the so-called sacral nucleus of Stilling. The lecturer gave an account of a patient under his treatment, in whom the disease had supervened, four years ago, owing to a severe chill; anæsthesia of the lower parts of the buttocks, the perinæum, the external and internal sexual organs, the urethra, and the bladder, and complete paralysis of the bladder and rectum were first noticed. Contraction could neither in a reflective way nor by voluntary impulse be produced. The reflective centre of the bladder and its connection with the brain had, therefore, been destroyed. When the effect of the anæsthesia in the present case was taken into consideration, it became evident that it was quite identical with the region which Luschka had described as the pelvic division. These branches derived their origin from the inferior sacral nerves, and, moreover, conducted the motory nerves for the whole uro-genitary system. As no trace of paralysis or anæsthesia of the lower extremity was present in the patient, the lumbar spinal cord could not be affected; the seat of the affection was, therefore, to be sought for in the cone of the spinal cord. An affection of the sacral plexus, where the nerves already contained mixed fibres, was also to be excluded, owing to the want of motory appearances.

This case, which had been observed by the lecturer, together with those which had been observed by Kirchhoff, admitted of the conclusion that the ano-vesical centre was situated in the "conus terminalis" of the spinal cord.

Professor Rosenthal furthermore made some remarks on the therapy of this disease, and pointed out that the internal remedies, such as ergotine, strychnine, etc., were of no value, and that much better results were obtained by hydro-therapeutic procedures, such as "douches" on the sacral bone, and so on. He, moreover, recommended a better method of electrification, which consisted in the fact that the "anode" of the galvanic current was applied to the nape, and the "cathode" on the superior part of the sacral bone, the current being repeatedly changed ("reversed"). In this way distinct contractions of the bladder were already obtained with a proportionately slight intensity of the electric current.

In the discussion which arose on the subject Dr. Teleky directed attention to the fact that, in old people, isolated paralysis of the bladder might also occur, and that he just now had such a case under treatment.

Professor Rosenthal replied that these isolated paralyses were always to be explained by a peripheral cause.

Dr. Fellner confirmed by his own experiences the statements of Professor Rosenthal.

Professor von Dittel was also of the opinion that most of the isolated paralyses of the bladder in old people were of a peripheral character, and that they consisted in atrophic disturbance of the muscles of the bladder.

